# Whole-Transcriptome Sequencing of Knee Joint Cartilage from Kashin–Beck Disease and Osteoarthritis Patients

**DOI:** 10.3390/ijms25084348

**Published:** 2024-04-15

**Authors:** Lixin Han, Bolun Cheng, Wenming Wei, Li Liu, Shiqiang Cheng, Huan Liu, Yumeng Jia, Yan Wen, Feng Zhang

**Affiliations:** 1 Collaborative Innovation Center of Endemic Disease and Health Promotion for Silk Road Region, School of Public Health, Health Science Center, Xi’an Jiaotong University, Xi’an 710061, China; shaanxiyqfk@163.com (L.H.); boluncheng@xjtu.edu.cn (B.C.); wenmingwei@stu.xjtu.edu.cn (W.W.); liuli0624@stu.xjtu.edu.cn (L.L.); chengsq0701@stu.xjtu.edu.cn (S.C.); huan.liu@xjtu.edu.cn (H.L.); jiayumemg@mail.xjtu.edu.cn (Y.J.); wenyan@mail.xjtu.edu.cn (Y.W.); 2 Key Laboratory of Trace Elements and Endemic Diseases (Xi’an Jiaotong University), National Health and Family Planning Commission, Xi’an 710061, China; 3 Key Laboratory of Environment and Genes Related to Diseases (Xi’an Jiaotong University), Ministry of Education, Xi’an 710061, China

**Keywords:** Kashin–Beck disease, osteoarthritis, messenger RNA, long noncoding RNA, circular RNA

## Abstract

The aim of this study was to provide a comprehensive understanding of similarities and differences in mRNAs, lncRNAs, and circRNAs within cartilage for Kashin–Beck disease (KBD) compared to osteoarthritis (OA). We conducted a comparison of the expression profiles of mRNAs, lncRNAs, and circRNAs via whole-transcriptome sequencing in eight KBD and ten OA individuals. To facilitate functional annotation-enriched analysis for differentially expressed (DE) genes, DE lncRNAs, and DE circRNAs, we employed bioinformatic analysis utilizing Gene Ontology (GO) and KEGG. Additionally, using quantitative reverse transcriptase polymerase chain reaction (qRT-PCR), we validated the expression levels of four cartilage-related genes in chondrocytes. We identified a total of 43 DE mRNAs, 1451 DE lncRNAs, and 305 DE circRNAs in KBD cartilage tissue compared to OA (*q* value < 0.05; |log_2_FC| > 1). We also performed competing endogenous RNA network analysis, which identified a total of 65 lncRNA-mRNA interactions and 4714 miRNA-circRNA interactions. In particular, we observed that *circRNA12218* had binding sites for three miRNAs targeting *ACAN*, while *circRNA12487* had binding sites for seven miRNAs targeting *COL2A1*. Our results add a novel set of genes and non-coding RNAs that could potentially serve as candidate diagnostic biomarkers or therapeutic targets for KBD patients.

## 1. Introduction

Kashin–Beck disease (KBD) is an endemic chronic osteochondropathy that predominantly affects children aged 3 to 12 years and subsequently becomes symptomatic with age [1]. The initial pathological changes of KBD occur in the deep zone of the growth plate cartilage and articular cartilage, resulting in premature closure of the epiphyseal plate, impaired endochondral ossification, multiple joint deformities, and skeletal growth retardation [2]. Osteoarthritis (OA) is a chronic disease affecting the joint and its tissues, primarily leading to progressive damage to articular cartilage and, subsequently, to the subchondral bone and surrounding synovial structures [3]. OA pathologically manifests as degradation of articular cartilage, subchondral bone sclerosis, and osteophyte formation [4]. The etiology and molecular mechanisms underlying KBD differ from those associated with OA [5], and elucidating the differences between these two diseases may provide valuable insights for pathogenetic and therapeutic studies of KBD.

Recent research has indicated that approximately 80% of the human genome is transcribed as noncoding RNAs (ncRNAs), which have been shown to manipulate numerous potential biological functions, including microRNAs (miRNAs), long noncoding RNAs (lncRNAs), and circular RNAs (circRNAs) [6,7]. Recent studies suggest that lncRNAs, circRNAs, pseudogenes, and mRNAs may function as miRNA sponges by competing for miRNA response elements [8,9]. This competition can modulate the progression of various degenerative diseases, such as OA. Several studies have reported aberrant expression patterns of lncRNAs and miRNAs in KBD. For example, a network based on lncRNA–miRNA–mRNA was constructed between KBD and healthy individuals to reveal several potential causative molecular and signaling pathways involved in KBD [10]. Similarly, an investigation into the intricate regulatory mechanisms and interactions between transcripts has linked ncRNAs to OA development [11]. An increasing number of ncRNAs have been identified for their important roles in both KBD and OA pathogenesis. Therefore, it is essential to conduct an integrative analysis between KBD and OA rather than studying them individually.

Over the past decade, an increasing number of studies have employed RNA sequencing (RNA-seq) or microarray techniques to elucidate the biological mechanisms underlying KBD. Wang et al. conducted mRNA expression profiling in articular cartilage from four pairs of age-matched patients diagnosed with KBD and OA, revealing significant differences in gene expression levels related to metabolism, apoptosis, cell proliferation, and matrix degradation activity [12]. Yang et al. utilized RNA-seq and miRNA array analysis to identify a total of 179 differently expressed genes (DEGs) and 124 differently expressed miRNAs (DEMs) in subchondral bone between KBD and OA, suggesting distinct pathological mechanisms in this region [13]. Furthermore, Wu et al. performed miRNA expression profiling in blood specimens, identifying 18 common DEMs in KBD compared to OA [14]. In another investigation, researchers examined the lncRNA expression profile in chondrocytes derived from KBD patients and healthy controls, implicating abnormal lncRNAs as key regulators of cartilage extracellular matrix degradation [15]. Additionally, using circRNA chips, Wang et al. identified 1627 differently expressed (DE) circRNAs between OA and KBD, highlighting *hsa_circRNA_0020014* as a potential biomarker for differential diagnosis [16]. Expanding the scope of analysis, Wang et al. employed single-cell RNA-seq, revealing a marked expansion of homeostatic and mitochondrial chondrocyte populations in KBD, characterized by the expression of unknown markers *MT1X*, *MT2A*, *MT-ND1*, and *MT-ATP6* [17]. However, despite the insights gained from independent RNA studies, a comprehensive understanding of disease transcriptional regulation in KBD and OA remains elusive. The interplay between lncRNA/circRNA-miRNA-mRNA regulatory mechanisms in these conditions requires further investigation at the whole-transcriptome level to gain deeper insights into their respective etiologies.

In this study, we utilized whole-transcriptome sequencing to comprehensively evaluate the expression patterns of mRNAs, lncRNAs, and circRNAs in knee joint cartilage specimens obtained from KBD and OA patients. The primary objective was to elucidate the transcript regulation features of non-coding RNAs underlying the pathogenesis of KBD compared to OA. Additionally, Gene Ontology (GO) and Kyoto Encyclopedia of Genes and Genomes (KEGG) pathway analyses were conducted to identify key biological functions associated with KBD.

## 2. Results

### 2.1. Descriptive Characteristics of Patients with KBD and OA

**Figure 1:**
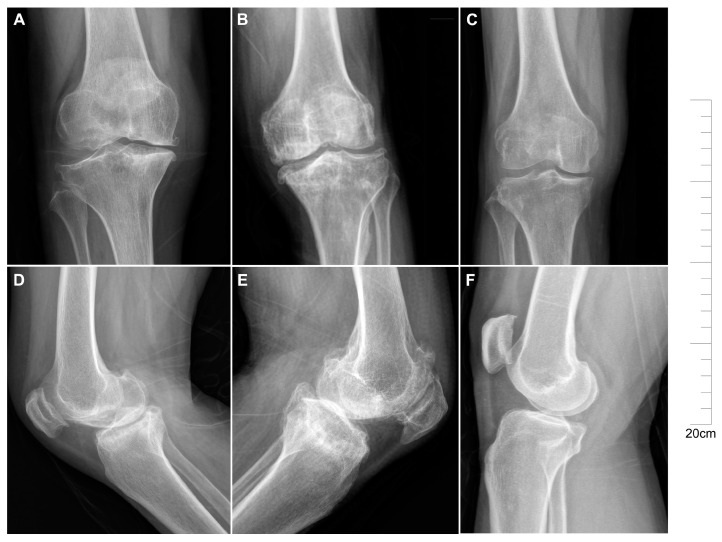
Characteristics of knee joint damage in samples of Kashin–Beck disease and osteoarthritis. Frontal X-ray of the knee joint in KBD (**A**), OA (**B**), and normal control (**C**) subjects. Lateral X-ray of the knee joint in KBD (**D**), OA (**E**), and normal control (**F**) subjects.

Clinical manifestations of KBD patients typically include joint deformities affecting the hands, knees, ankles, and other areas. These symptoms are often accompanied by restricted mobility and even short stature. Radiological examinations reveal distinct features in KBD, such as blurred margins of metaphysis along with wide and deformed bone ends (Figure 1A,D). In comparison to normal control (Figure 1C,F), the manifestation of cartilage injury in KBD is typified by extensive areas of cartilage detachment, partial loss leading to bone exposure, and irregular bone surfaces (Figure 1A,D). Conversely, cartilage injury in OA is marked by cartilage erosion, with occasional remnants of cartilage islands at sites of bone exposure. The exposed bone surface is uneven and may exhibit fibrous bands adhering to its surface (Figure 1B,E). In comparison to OA patients, KBD patients demonstrate more severe cartilage damage in the knee joint. Despite both conditions ultimately resulting in significant joint damage, their pathological morphology differs.

### 2.2. Identification of DE mRNAs between KBD and OA

**Figure 2:**
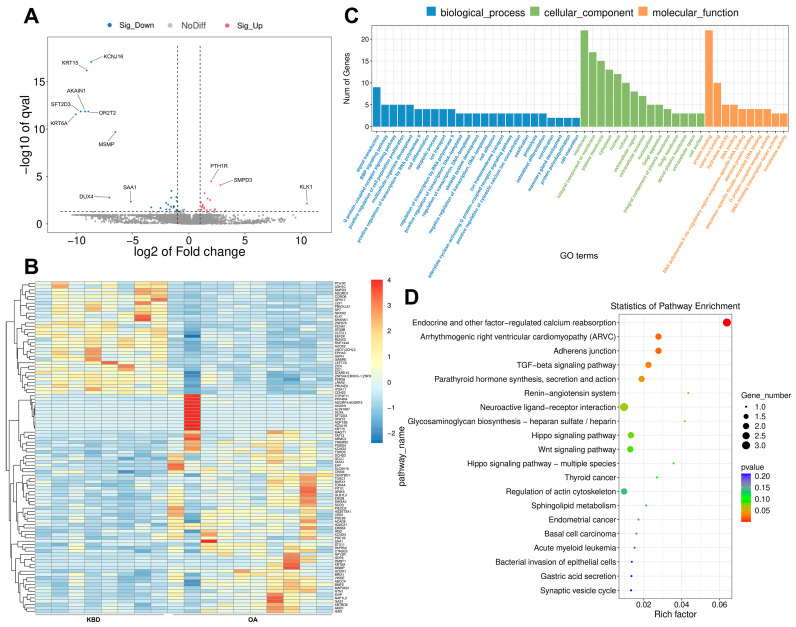
Overview of the mRNA expression profile. (**A**) Volcano plots for DE mRNA (KBD vs. OA). The gray dotted line indicates the threshold of significant genes, the horizontal line (−log_10_ 0.05) and the vertical line (log_2_ 2). (**B**) A heatmap is used to assess the expression of mRNA. The color scheme uses red to denote high expression and blue to indicate low expression. Each DE mRNA is represented by a single row of colored boxes, while each sample is represented by a single column. (**C**) GO-term analysis of DE mRNA. (**D**) KEGG analysis of DE mRNA.

To assess mRNA expression levels in KBD and OA, a total of 18 mRNA libraries were constructed. Compared with OA, 43 genes, including 18 upregulated genes and 25 downregulated genes, were differentially expressed in KBD (*q* value < 0.05; |log_2_FC| > 1) (Figure 2A and Supplementary Table S1). Hierarchical cluster analysis suggested that some DEGs may be involved in the regulation of the same pathway or possess comparable functions (Figure 2B). In order to further explore the functional implications of these DEGs, GO and KEGG pathway analyses were performed. A total of 189 significantly enriched GO terms were identified with *p* values < 0.05 (Figure 2C and Supplementary Table S2), wherein approximately 72.5%, 8.5%, and 19.0% mRNAs were assigned to biological process (BP), cellular component (CC), and molecular function (MF), respectively. The BP category was found to be associated with the BMP signaling pathway, osteoblast development, and chondrocyte development. The MF category demonstrated associations with collagen receptor activity, collagen binding involved in cell-matrix adhesion, and glutamate receptor activity. The CC category exhibited associations with the integrin alpha11-beta1 complex, coated vesicle, and kainate selective glutamate receptor complex. Furthermore, the DEGs were aligned within the KEGG pathway database. As shown in Figure 2D and Supplementary Table S3, the pathways of endocrine and other factor-regulated calcium reabsorption, arrhythmogenic right ventricular cardiomyopathy (ARVC), adherens junction, and TGF-β signaling pathways were mainly activated (*p* value < 0.05).

### 2.3. Prediction and Function Analysis of the lncRNA Target Genes

**Figure 3:**
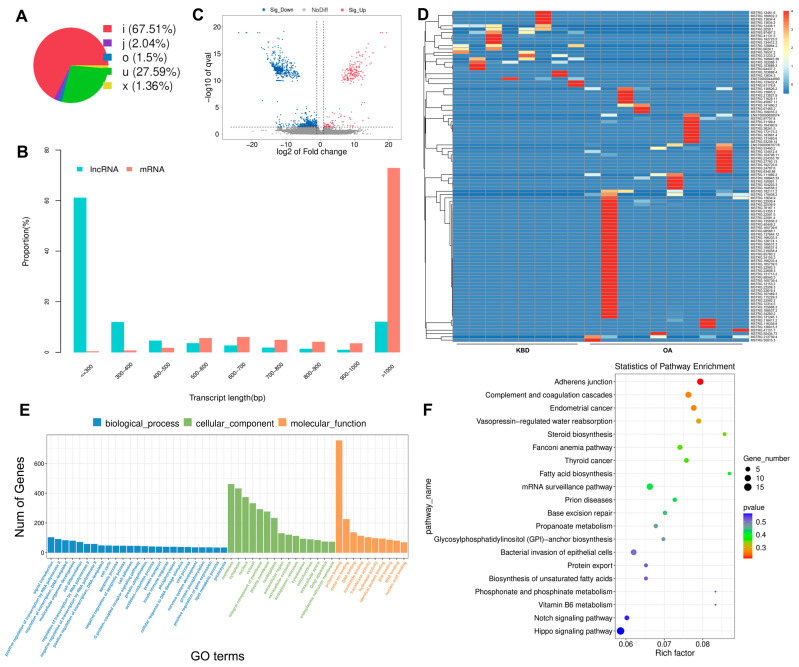
Prediction and function analysis of lncRNA target genes. (**A**) Distribution of the lncRNA type. i, a transcribed fragment falling entirely within a reference intron; j, potentially novel isoform (transcribed fragments): at least one splice junction is shared with a reference transcript; o, generic exonic overlap with a reference transcript; u, unknown, intergenic transcript; x, exonic overlap with reference on the opposite strand. (**B**) Comparison of the length of lncRNA and mRNA density distribution. The proportion was calculated based on the number of transcripts falling within each length category. (**C**) Volcano plots for DE lncRNA. The gray dotted line indicates the threshold of significant genes, the horizontal line (−log_10_ 0.05) and the vertical line (log_2_ 2). (**D**) A heatmap is used to assess the expression of lncRNA. (**E**) GO analysis of target genes of DE lncRNA. (**F**) KEGG analysis of target genes of DE lncRNA.

The novel lncRNAs were classified into five types based on their positional relationship with known mRNAs (Figure 3A). To ensure that the novel lncRNA met the general characteristics, we compared its transcript length to that of mRNA (Figure 3B). Our analysis revealed that about 40% of the novel lncRNA adhered to typical transcript characteristics (transcripts with a length > 300 bp) (Figure 3B). The DE lncRNA expression patterns between KBD and OA were also examined, including 170 up-regulated and 1281 down-regulated lncRNAs (Figure 3C and Supplementary Table S4). Target gene prediction was performed for all identified lncRNAs, followed by functional enrichment analysis using GO/KEGG methodologies to predict their main biological functions. A total of 41 GO terms were extracted (Figure 3E and Supplementary Table S5). In the BP category, regulation of ion transport, strand invasion, and response to glucoside were functionally enriched. The CC category demonstrated association with the juxtaparanode region of the axon, an anchored component of the postsynaptic membrane. The MF category was found to be associated with RAGE receptor binding, ion channel regulator activity, sulfate binding, and UMP kinase activity. However, no significant KEGG pathway was enriched by the DE lncRNA (Figure 3F and Supplementary Table S6).

### 2.4. Overview of the circRNA Sequencing Data

**Figure 4:**
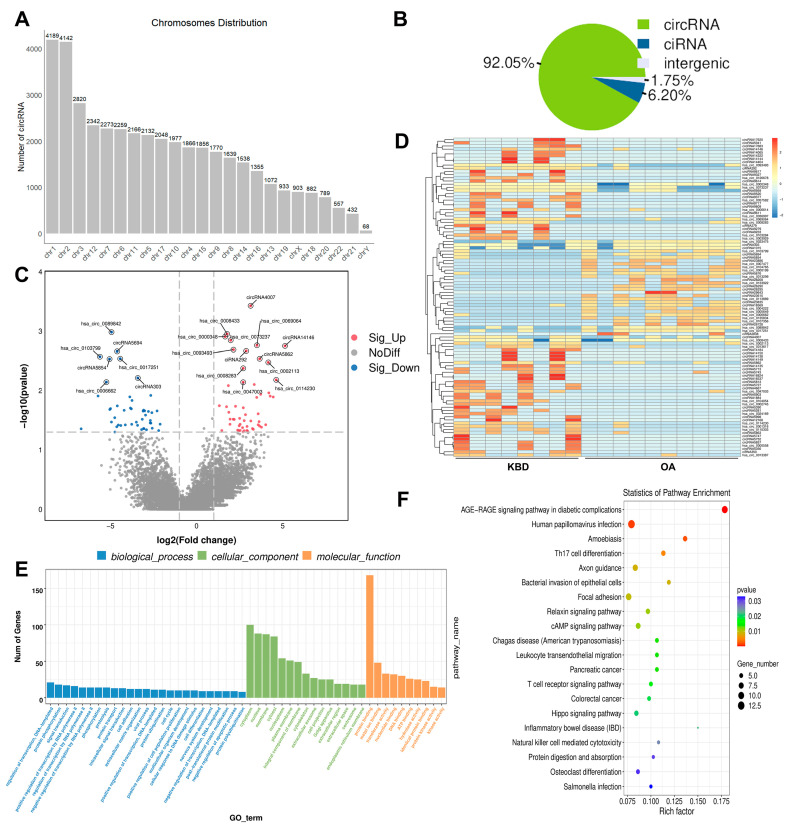
Identification and function analysis of circRNA. (**A**) Genome distribution of circRNAs. (**B**) The source of circRNA. (**C**) Volcano plots for DE circRNA. The gray dotted line indicates the threshold of significant genes, the horizontal line (−log_10_ 0.05) and the vertical line (log_2_ 2). (**D**) A heatmap is used to assess the expression of circRNA. (**E**) GO-term analysis of circRNA. (**F**) KEGG analysis of DE circRNA.

In order to elucidate the roles of circRNAs in KBD compared to OA, we conducted circRNA sequencing using the rRNA-depleted cartilage tissue obtained from individuals with KBD and OA. A total of 42,297 circRNAs were identified and found to be widely distributed across all chromosomes (Figure 4A). Notably, our analyses revealed that the majority of these circRNAs (92.05%) were derived from exonic regions, while only a small proportion (6.21% and ~1.75%) originated from intronic and intergenic regions, respectively (Figure 4B). To identify DE circRNAs that are potentially involved in KBD, we compared their expression patterns between KBD and OA. A total of 305 DE circRNAs were observed, including 184 upregulated and 121 downregulated genes (Figure 4C and Supplementary Table S7). To gain further insight into the biological functions of these DE circRNAs, we conducted GO and KEGG functional analyses. Specifically, GO analysis identified 223 terms within the BP category, such as the regulation of immune response, tissue morphogenesis, and the integrin-mediated signaling pathway. The dominant functions in each of the remaining two categories included extracellular matrix and collagen-containing extracellular matrix in the CC category and platelet-derived growth factor binding and extracellular matrix structural constituents in the MF category (Figure 4E and Supplementary Table S8). In addition, KEGG pathways analysis revealed that the pathways of the AGE-RAGE signaling pathway in diabetic complications, human papillomavirus infection, and amoebiasis were activated in the function of these circRNAs (Figure 4F and Supplementary Table S9). Supplementary Tables S10 and S11 summarized distinct and common GO terms and KEGG pathways enriched among DE mRNAs, DE IncRNAs, and DE cricRNAs, respectively.

### 2.5. Construction of the Potential lncRNA-mRNA and circRNA-miRNA Interactions

To further clarify the functional relationship between lncRNA and its target mRNA, as well as circRNA and its target miRNA, we conducted a comprehensive analysis of the interaction. For lncRNA-mRNA, 65 interactions were identified with 37 lncRNA and 54 mRNA, respectively (Supplementary Table S12). Notably, differences in these lncRNA-mRNA interactions for KBD compared to OA were associated with immune response and transcription regulatory region sequence-specific DNA binding. For miRNA-circRNA, 4714 interactions were identified with 200 miRNAs and 295 circRNAs, respectively (Supplementary Table S13). Notably, differences in circRNA and miRNA interactions for KBD compared to OA were associated with target marker genes regulating cartilage growth. For example, *circRNA12218* had binding sites for three miRNAs (*hsa-miR-132-5p*, *hsa-miR-205-3p*, and *hsa-miR-221-5p*), which target *ACAN* (ENST00000352105). *CircRNA12487* had binding sites for seven miRNAs (*hsa-miR-106a-5p*, *hsa-miR-147a*, *hsa-miR-17-5p*, *hsa-miR-204-3p*, *hsa-miR-20a-5p*, *hsa-miR-214-3p*, and *hsa-miR-93-5p*), which target *COL2A1* (ENST00000337299). 

### 2.6. Validation of Differentially Expressed mRNAs and ncRNAs in Human

**Figure 5:**
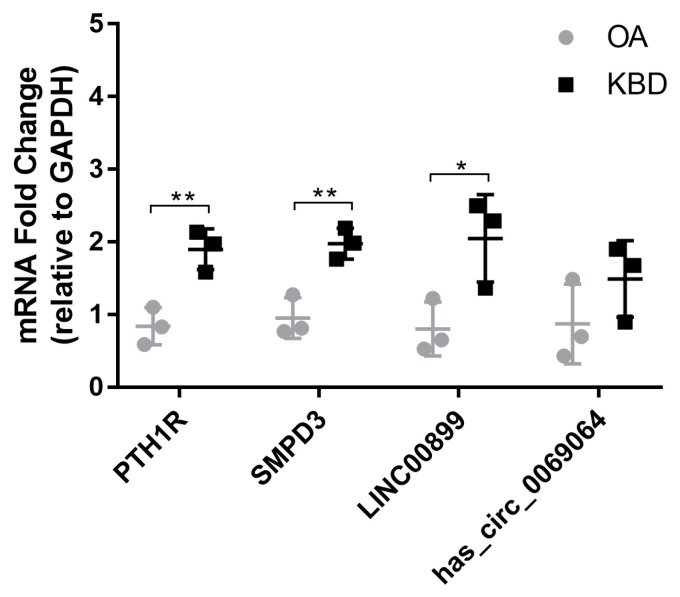
Confirmation of the differential expression of mRNAs and ncRNAs. mRNA fold change for *PTH1R*, *SMPD3*, *LINC00899*, and *hsa_circ_0069064* in chondrocyte, measured by qRT-PCR with triplicate replication; symbols represent each individual cartilage donor; bars show the mean ± SD; OA, osteoarthritis; KBD, Kashin–Beck disease. * *p* value < 0.05, ** *p* value < 0.01.

To validate our sequencing results, chondrocyte samples were collected from three KBD and three OA patients, and qRT-PCR was performed for *PTH1R*, *SMPD3*, *LINC00899*, and *hsa_circ_0069064* verification. The results of qRT-PCR were consistent with those of Illumina sequencing (Figure 5), which might partly confirm the accuracy and reliability of our initial sequencing data.

## 3. Discussion

Whole transcriptome sequencing is a powerful tool that can provide insights into the regulatory relationships between various ncRNAs and mRNAs and the complex interaction network between ncRNAs. In this study, we conducted whole transcriptome sequencing to analyze cartilage tissue obtained from KBD and OA patients. Our comprehensive analysis revealed 43 DE mRNAs, 1451 DE lncRNAs, and 305 DE circRNAs. The biological functions of the above RNAs were subsequently analyzed by GO and KEGG enrichment analyses. Our results represent an important resource for advancing the understanding of ncRNAs during disease progression in KBD patients.

Selenium deficiency emerges as a significant epidemiological characteristic of KBD. In 1972, Mo et al. observed a marked decrease in selenium content in both water and grain within KBD-affected regions compared to non-affected areas [18]. The geographical distribution of KBD aligns notably with regions characterized by low selenium levels [19]. Most epidemiological studies corroborate this observation, noting lower selenium levels in both environmental samples and human populations within KBD-affected areas compared to unaffected regions. Nonetheless, it is worth noting that certain affected regions exhibit selenium levels comparable to non-affected areas [20]. While the exact etiology of KBD remains elusive and selenium deficiency alone may not be its direct cause, it serves as a significant environmental risk factor.

Our analysis identified multiple DEGs that may be associated with cartilage development and selenium deficiency, such as *PTH1R* and *SAA1*. Of particular interest is the role of parathyroid hormone-related peptide (PTHrP), which has been shown to prevent premature chondrocyte differentiation by activating parathyroid hormone/PTHrP receptor 1 (PTH1R), the only known receptor for the PTHrP ligand [21]. Parathyroid hormone-related peptide (PTHrP) is predominantly expressed within the prehypertrophic zone, situated between the proliferating and hypertrophic zones [22]. PTHrP serves to stimulate both proliferation and hypertrophy of chondrocytes within these zones, consequently delaying the terminal differentiation of chondrocytes during the process of endochondral bone development [23]. The impact of combined selenium and iodine deficiency on bone and cartilage growth is noteworthy. It has been observed that the alterations in *PTHrP* expression induced by the combined deficiency of selenium and iodine align with the measurements of *PTHrP* in KBD [24]. Previous studies have demonstrated that evaluating markers of different stages of chondrocyte differentiation can provide insights into the role played by PTH1R signaling in experimental degenerative articular cartilage using genetic mouse models [25]. Additionally, other studies have reported that *PTH1R* can regulate the terminal differentiation process in deep-zone chondrocytes [26] and inhibit IL-1β-induced chondrocyte hypertrophy [27]. Changes in serum amyloid A1 (SAA1) protein levels have been associated with inflammation and immune pathway alterations observed in patients with knee OA, and treatment targeting *SAA1* expression may improve pain sensitivity in adults with knee OA [28]. Despite these findings, the specific regulatory mechanisms by which lncRNAs may modulate gene expression during the progression of KBD remain unclear and require further investigation. 

LncRNAs represent a highly heterogeneous class of RNAs that can be transcribed from various regions of protein-coding genes and intergenic regions in either sense or antisense orientation. This study identified several DE lncRNAs related to cartilage damage, such as *CYTOR* and *MEG8*. Notably, numerous lncRNAs have been implicated in articular cartilage degradation, underscoring the potential importance of these regulatory molecules in disease progression. Specifically, cytoskeleton regulator RNA (*CYTOR*), also known as *LINC00152,* is an 828-nucleotide-long lncRNA that has been shown to exhibit abnormal expression patterns across multiple complex diseases [29]. It is reported that *CYTOR* is a pivotal lncRNA in modulating age-related articular cartilage degradation, and *CYTOR* expression was found to be significantly reduced in cartilage tissues from OA patients [30]. Integration analysis of bulk RNA-seq and single-cell RNA-seq data from OA chondrocytes has indicated that lncRNA regulation between *CYTOR* and *NRP1* may be involved in pain and vascularization associated with cartilage degeneration in OA knees [31]. Overexpression of *CYTOR* has been shown to promote chondrocyte viability while simultaneously suppressing their apoptosis and inflammatory response [32]. Maternally expressed 8 (*MEG8*), a nuclear-localized lncRNA, has been demonstrated to exert protective effects during the progression of OA [33]. Notably, the attenuation of *MEG8* expression significantly impeded proliferation, induced cell death, and provoked inflammatory responses in chondrocytes [34]. Despite these findings, the specific regulatory mechanisms by which lncRNAs may modulate gene expression during the progression of KBD remain unclear and require further investigation.

A single circRNA has the potential to associate with multiple identical miRNAs. In our investigation of circRNA-miRNA networks relevant to KBD, we observed that circRNA and lncRNA may exert a central regulatory role. Notably, a single circRNA or lncRNA can interact with numerous miRNAs as well as mRNAs. For example, *hsa_circ_0004662* was found to have binding sites for 11 miRNAs (*hsa-miR-129-5p, hsa-miR-135a-5p*, *hsa-miR-148a-3p*, *hsa-miR-15a-5p*, *hsa-miR-15b-5p*, *hsa-miR-16-5p*, *hsa-miR-196a-5p*, *hsa-miR-198*, *hsa-miR-208a-5p*, *hsa-miR-30a-3p*, *hsa-miR-30d-3p*). Previous studies have reported that *hsa_circ_0004662* can promote the progression of OA through modulation of the *miR-424-5p*/*VEGFA* axis [35]. Specifically, knockdown of *hsa_circ_0004662* resulted in increased chondrocyte proliferation, decreased apoptosis and expression levels of *Bax* and *MMP13*, as well as increased *ACAN* and *BCL2* expression in IL-1β-induced chondrocytes [35]. These effects were subsequently reversed by further down-regulation of miR-424-5p or overexpression of *VEGFA* [35]. 

GO enrichment analysis revealed that chondrocyte-associated biological processes were encompassed by numerous differentially expressed RNAs in KBD, such as positive regulation of chondrocyte differentiation and extracellular matrix, which was consistent with recent findings in KBD and normal control samples [10]. KEGG pathway enrichment analysis indicated that DE RNAs in KBD were associated with various pathways such as adherens junction, TGF-β signaling pathway, parathyroid hormone synthesis, secretion and action, human papillomavirus infection, and osteoclast differentiation. These findings are partly consistent with the previous RNA-seq study utilizing articular cartilage isolated from KBD and rheumatoid arthritis patients [36]. KBD and OA exhibit not only similar clinical characteristics but also share associated pathologic articular cartilage disorders, characterized by chondrocyte apoptosis, inflammation, and cartilage degeneration [37]. Our results underscore the commonalities between KBD and OA regarding the manifestation and pathological changes in articular cartilage. Our GO analysis has elucidated pathways such as the BMP pathway, osteoblast development, and chondrocyte development, indicating shared mechanisms of cartilage remodeling—a process fundamental to both KBD and OA. Moreover, several common pathways related to OA pathogenesis were also identified. For instance, the TGF-β signaling pathway emerged as one of the common pathways. This pathway is crucial for maintaining cartilage homeostasis [38], and inhibition of TGF-β signaling in mesenchymal stem cells beneath the cartilage can alleviate osteoarthritis symptoms [39]. Similarly, the amoebiasis pathway showed overlap with our findings. A recent proteomic analysis of total bone protein in primary knee osteoarthritis patients identified proteins with differential N-glycosylation sites enriched in metabolic pathways such as amoebiasis [40].

The competing endogenous RNAs (ceRNAs) play a significant role in the regulation of gene expression [41]. In this study, we systematically analyzed the differences in gene expression and regulation of non-coding RNA and constructed circRNA-miRNA networks for KBD based on our RNA-seq data. There are several limitations to the present study. Firstly, it is important to note that the sample size was relatively small. Secondly, the potential technical limitations, such as platform-specific biases, variations in RNA extraction methods, and potential batch effects, might subsequently influence the accuracy and reproducibility of our results. Thirdly, the predicted lncRNA-mRNA interactions and miRNA-circRNA interactions should be interpreted with caution due to the lack of experimental validation. Fourth, KBD is primarily attributed to the degeneration of growth plate cartilage cells during childhood or early adolescence, resulting in progressive joint deformities. This process complicates the determination of the age at which individuals develop KBD deformities. In addition, several potential future directions should be considered. To ensure robustness and generalizability of our findings, an additional validation cohort should be included in sub-sequent studies to comprehensively analyze the expression patterns of identified mRNAs, lncRNAs, and circRNAs. This will allow us to accurately estimate risk ratios associated with candidate genes and identify KBD biomarkers that can be effectively utilized in clinical diagnostic and treatment applications. Moreover, while our study provides valuable insights into the interactions between circRNA and miRNA axes within KBD pathology, further experimental investigations are required to validate these interactions and fully elucidate their underlying mechanisms within KBD pathogenesis. In addition, encouraging research focused on exploring specific molecular targets implicated in shared pathways between KBD and other OA conditions, with the goal of developing targeted therapeutic interventions.

Developing RNA molecules as biomarkers for these complex diseases presents several significant challenges. Firstly, deciphering the intricate interplay among non-coding RNAs represents a significant objective. Nonetheless, conducting comprehensive high-throughput profiling of both joint tissues and biofluids, essential for attaining this goal, poses unique challenges [42]. Additionally, the heterogeneity of OA and KBD poses a challenge, as the diseases can manifest differently in various individuals, necessitating the identification of biomarkers capable of capturing this variability. Furthermore, the dynamic nature of RNA expression profiles presents challenges in selecting stable and consistent biomarkers over time [43], especially considering the progressive nature of OA and the fluctuating disease course of KBD. Another challenge lies in the standardization of sample collection, RNA extraction, and analysis protocols to minimize variability and ensure reproducibility across studies [44]. Moreover, the translation of RNA biomarkers from research settings to clinical applications requires addressing regulatory and logistical hurdles, including obtaining regulatory approvals, developing cost-effective diagnostic assays, and integrating biomarker testing into existing healthcare frameworks [45]. Despite these challenges, the potential of RNA molecules as biomarkers for KBD holds promise for advancing early detection, prognostication, and therapeutic monitoring.

## 4. Materials and Methods

### 4.1. Ethic Statement

The present study was approved by the Human Ethics Committee of Xi’an Jiaotong University. Written informed consent was obtained from all study participants. 

### 4.2. Patients and Samples

The present investigation involved the collection of cartilage specimens from the knee joints of patients diagnosed with KBD and OA who were undergoing total knee joint arthroplasty. KBD donors were obtained from Yongshou County, Xi’an City, Shaanxi Province, while OA donors were sourced from Xi’an City within the same province. All study subjects were Chinese Han individuals who underwent a thorough clinical examination and radiography assessment prior to inclusion. General information regarding each participant was collected via a nurse-administered questionnaire, including self-reported ethnicity, lifestyle characteristics, health status, and medical history. The diagnosis of KBD was based on established criteria outlined in the KBD diagnosis criterion of China (WS/T 207-2010) [46], which includes clinical features such as enlarged bone ends of phalanges, shortened phalanges, and joint deformities. Patients with primary knee OA were diagnosed based on the Kellgren/Lawrence (K/L) method [47], clinical evaluation, and radiologic imaging. Individuals with a history of genetic bone or cartilage disorders, rheumatoid arthritis, or a family history of other articular diseases were excluded from our study. Cartilage samples were meticulously collected to prevent contamination of bone, synovium, or blood components and subsequently stored in liquid nitrogen for further analysis. Finally, eight patients diagnosed with KBD (two males and six females) and ten patients diagnosed with OA (three males and seven females) were selected for whole-transcriptome sequencing analysis. The average ages of KBD and OA participants were 61.75 ± 3.73 and 62.00 ± 4.40 years old, respectively. The clinical information of KBD and OA patients is summarized in Supplementary Table S14.

### 4.3. RNA Library Construction and Sequencing

Total RNA was extracted and purified using Trizol reagent (Invitrogen, Carlsbad, CA, USA) in accordance with the manufacturer’s instructions. The quantity and purity of each RNA sample were determined using a NanoDrop ND-1000 spectrophotometer (NanoDrop, Wilmington, DE, USA), while RNA integrity was assessed by an Agilent 2100 (Agilent Technologies, Palo Alto, CA, USA) with an RIN number greater than 7.0. Approximately 5 µg of total RNA was utilized to deplete ribosomal RNA via the Ribo-Zero™ rRNA Removal Kit protocol (Illumina, San Diego, CA, USA). Following the depletion of ribosomal RNAs from the samples, the remaining RNAs were fragmented into smaller pieces via treatment with divalent cations under high-temperature conditions. These cleaved fragments were then reverse-transcribed to generate complementary DNA (cDNA), which subsequently served as templates for the synthesis of U-labeled second-stranded DNAs utilizing *E. coli* DNA polymerase I along with RNase H and dUTP. To prepare each strand for ligation to indexed adapters, an A-base is added to the blunt ends of DNA fragments. Each adapter contains a T-base overhang for ligating the adapter to the A-tailed fragmented DNA. Single- or dual-index adapters are then ligated onto the fragments, and size selection is performed using AMPureXP beads. After treatment with the heat-labile UDG enzyme, U-labeled second-stranded DNAs undergo PCR amplification under conditions consisting of initial denaturation at 95 °C for 3 min, 8 cycles of denaturation at 98 °C for 15 s, annealing at 60 °C for 15 s, extension at 72 °C for 30 s, and concluding with final extension at 72 °C for 5 min. The average insert size in the final cDNA library was 300 bp (±50 bp). Finally, paired-end sequencing was conducted on an Illumina Hiseq 4000 (Illumina, Inc., San Diego, CA, USA) following the vendor’s recommended protocol.

### 4.4. Preprocessing for Whole RNA Sequencing Data 

Initially, Cutadapt (version 3.0) [48] was employed to eliminate the reads containing adaptor contamination, low-quality bases, and undetermined bases. Subsequently, FastQC (version 0.12.0, http://www.bioinformatics.babraham.ac.uk/projects/fastqc/, accessed on 1 June 2023) was utilized for sequence quality verification. Bowtie2 (version 2.5.2) [49] and Hisat2 (version 2.2.0) [50] were used to map reads onto the genome of human hg38. The mapped reads of each sample were assembled using StringTie (version 2.2.0) [51]. Perl scripts were then utilized to merge all transcriptomes obtained from samples into a comprehensive transcriptome. After generating the final transcriptome dataset, expression levels of all transcripts were estimated via StringTie (version 2.2.0) [51] and edgeR (version 4.0.16) [52].

### 4.5. Differential Expression Analysis of mRNAs and lncRNAs

Initially, transcripts shorter than 200 bp and those that overlapped with known mRNAs were excluded from analysis. Next, the Coding Potential Calculator (CPC, version 0.9-r2) [53] and Coding-Non-Coding Index (CNCI, version 2.0) [54] algorithms were employed to predict transcripts with coding potential. Transcripts with a CPC score < −1 and a CNCI score < 0 were eliminated, while those without any coding potential were considered lncRNAs. StringTie (version 2.2.0) [51] was used to perform expression levels for mRNAs and lncRNAs by calculating FPKM [55]. Differential expression analysis was conducted utilizing the R package edgeR (version 4.0.16) [52] to identify differentially expressed mRNAs and lncRNAs based on log_2_ (fold change) > 1 or log_2_ (fold change) < −1 along with FDR statistical significance (*q* value < 0.05).

### 4.6. Potential Target Gene Prediction and Functional Analysis of lncRNAs

In order to investigate the potential function of lncRNAs, we comprehensively predicted the cis-target genes of lncRNAs. It is well established that lncRNAs can exert cis-regulatory effects on neighboring target genes. In this study, we employed a Python script to select coding genes located within 100,000 base pairs upstream and downstream of the lncRNA loci under investigation. We limited our analysis to predicting cis interactions within a range of 100 kb upstream and downstream of the DE lncRNAs. The potential target genes of lncRNAs were predicted using Starbase 2.0 software [56] and the MEM database [57]. Subsequently, we showed functional annotation of the target genes for lncRNAs was performed using BLAST2GO (version 6.0) [58]. Statistical significance was determined by setting the threshold for a *p* value < 0.05.

### 4.7. Prediction, Annotation, and miRNA Interaction of Novel circRNAs

The reads containing adaptor contamination, low-quality bases, and undetermined bases were eliminated using Cutadapt (version 3.0) [48]. Subsequently, sequence quality was evaluated with FastQC (version 0.12.0, http://www.bioinformatics.babraham.ac.uk/projects/fastqc/, accessed on 3 June 2023). Bowtie2 (version 2.5.2) [49] and Hisat2 (version 2.2.0) [50] were used to map reads to the genomes of species. The tophat fusion [59] was used for unmapped reads for additional mapping to the genome. CIRCExplorer2 [60,61] and CIRI [62] were used for de novo assembly of mapped reads into circRNAs. In addition, back-splicing reads were identified in unmapped reads through the utilization of the tophat-fusion algorithm. Each sample produced a unique set of circRNAs that underwent further analysis for differential expression via R package-edgeR (version 4.0.16) [52]. The DE circRNAs were selected based on log_2_ (fold change) > 1 or log_2_ (fold change) < −1 along with FDR statistical significance (*q* value < 0.05). The relationship between circRNAs and miRNAs was then predicted by using TargetScan (version 7.0) and miRanda (version 0.10.80) software [63].

### 4.8. Functional Enrichment Analysis

In order to gain further insights into the functional implications of DE miRNAs and DE circRNAs, we performed Gene Ontology (GO) enrichment analysis on the target gene candidates associated with these molecules. Furthermore, we utilized the KEGG Orthology-Based Annotation System software (version 2.0)_to assess the statistical enrichment of these target genes within KEGG pathways. GO terms and KEGG pathways that exhibited a *p* value < 0.05 were considered significantly enriched by differential expression of genes in our study.

### 4.9. Validation of the Differential Expression of mRNAs, lncRNAs, and circRNAs

To validate the findings of our transcriptome sequencing, we conducted real-time PCR to determine the RNA levels of two mRNAs, one lncRNA, and one circRNA in chondrocytes [64,65]. The RNA was extracted and isolated from chondrocytes using the TRIzol reagent (Invitrogen Ltd., CarIsbad, ON, Canada) [66]. Subsequently, 500 ng of the isolated RNA underwent reverse transcription to synthesize cDNA using a one-step kit (TaKaRa PrimeScriptTM RT reagent Kit, Beijing, China). Real-time PCR was carried out on the CFX96 Real-Time PCR System (Bio-Rad Laboratories, Inc., Hercules, CA, USA), utilizing 2× SYBR Green Mix as the detection method. The amplification procedure involved an initial pre-degeneration step of 30 s at 95 °C, followed by 40 cycles consisting of 30 s at 95 °C and a subsequent annealing/extension phase for 34 s at 60 °C. The average Ct value was utilized to calculate the relative expression levels of target genes through the comparative 2^−ΔΔCt^ method, with glyceraldehyde-3-phosphate dehydrogenase (*GAPDH*) serving as the reference gene [67]. The PCR primer sets for *PTH1R*, *SMPD3*, *LINC00899*, *hsa_circ_0069064,* and *GAPDH* were obtained from Takara Bio (Supplementary Table S15). All the qRT-PCR experiments were conducted in triplicate to ensure reproducibility and accuracy.

### 4.10. Statistical Analysis

Two groups with normally distributed data were compared using *t* tests. All statistical analyses were performed using R software (version 4.3.0). All results are presented as means ± standard deviation (SD), and a *p* value < 0.05 or FDR (*q* value) < 0.05 was considered statistically significant in our study.

## 5. Conclusions

In summary, we analyzed the expression of ncRNAs and explored the interaction between circRNA and miRNA in KBD and OA cartilage. Our study revealed that KBD and OA share similar pathological features. Furthermore, we identified a range of ncRNAs, including lncRNAs and circRNAs, in KBD cartilage that exhibited significant differential expression compared to those observed in OA cartilage. While further experimental validation is necessary, these DE mRNA, circRNA, and lncRNA profiles have the potential to enhance our understanding of fundamental characteristics inherent to human knee cartilage tissue. Moreover, they may serve as diagnostic biomarkers or therapeutic targets for KBD patients distinct from those with OA.

## Data Availability

The data presented in this study are available on request from the corresponding author (fzhxjtu@mail.xjtu.edu.cn).

## Supplementary Materials

The supporting information can be downloaded at: https://www.mdpi.com/article/10.3390/ijms25084348/s1.
