# Supplementary material for: Whole-Transcriptome Sequencing of Knee Joint Cartilage from Kashin–Beck Disease and Osteoarthritis Patients
Source: Int J Mol Sci. 2024 Apr 15;25(8):4348. doi: 10.3390/ijms25084348 (PMC11049856; doi:10.3390/ijms25084348)
Supplement: Supplementary file 1 [file ijms-25-04348-s001.zip › Supplementary Table S14.pdf]

**Supplementary Table S14. Characteristics of subjects enrolled in whole-transcriptome sequencing**

| KBD (n=8) | | | OA (n=10) | | |
|---|---|---|---|---|---|
| Age | Sex | Current Grade | Age | Sex | Current K/L Grade |
| 58 | Male | III | 65 | Male | III |
| 65 | Male | III | 65 | Male | III |
| 60 | Female | II | 68 | Male | III |
| 69 | Female | III | 61 | Female | III |
| 58 | Female | III | 62 | Female | III |
| 63 | Female | III | 61 | Female | II |
| 58 | Female | II | 59 | Female | II |
| 63 | Female | III | 64 | Female | III |
| / | / | / | 51 | Female | III |
| / | / | / | 64 | Female | III |

KBD, Kashin-Beck disease; OA, Osteoarthritis; K/L, Kellgren/Lawrence.
